# Application of large language models in early screening of Alzheimer’s disease: a scoping review

**DOI:** 10.3389/fnagi.2026.1906641

**Published:** 2026-10-12

**Authors:** Jianan Tian, Weidong Huang, Yongmei Wen, Yifan Liang

**Affiliations:** 1 School of Nursing, Changchun University of Chinese Medicine, Changchun, China; 2 The Affiliated Hospital of Changchun University of Chinese Medicine, Changchun, China

**Keywords:** Alzheimer’s disease, early screening, elder, large language models, scoping review

## Abstract

**Objective:**

To systematically map the current applications of large language models (LLMs) in the early screening of Alzheimer’s disease (AD) and to provide evidence to inform future research and nursing practice.

**Methods:**

This scoping review was conducted in accordance with the Joanna Briggs Institute (JBI) methodological framework. A comprehensive search was performed in PubMed, Web of Science, Embase, Cochrane Library, ProQuest, and Scopus from database inception to April 20, 2026. Eligible studies were screened, selected, and synthesized according to predefined inclusion criteria.

**Results:**

A total of 25 studies were included. The LLMs applied in these studies mainly consisted of general-purpose generative models, multimodal models, and medical-specific models. Data sources primarily included public databases, along with real-world clinical data and simulated data. LLMs were mainly applied for cognitive impairment identification, data augmentation, automated cognitive assessment, and clinical decision support, with cognitive impairment identification being the predominant application direction. Several studies employed prompting strategies including zero-shot prompting, few-shot prompting, chain-of-thought prompting, and agent-based prompting. Multimodal integration and agent-based collaborative reasoning have emerged as important research directions in recent years.

**Conclusion:**

Large language models provide a novel technological approach for early AD screening and demonstrate potential for supporting cognitive assessment and risk identification. Future research should strengthen real-world studies, multicenter validation, and the development of model safety and ethical governance frameworks to facilitate the standardized application of LLMs in early AD screening.

## 1 Introduction

Alzheimer’s disease (AD) is the most common neurodegenerative disorder and a leading cause of cognitive impairment and functional disability among older adults worldwide (Carrillo and Mahinrad, 2026). With ongoing population aging, the number of individuals living with AD continues to increase, imposing substantial burdens on patients, caregivers, healthcare systems, and society as a whole (Alzheimer’s Association, 2025). Given the absence of disease-modifying therapies capable of reversing the underlying pathological process, early identification of cognitive decline and timely intervention are widely recognized as critical strategies for delaying disease progression and improving clinical outcomes (Singh and Shukla, 2025). Currently, the early detection of AD primarily relies on conventional cognitive assessment instruments, including the Mini-Mental State Examination (MMSE) and the Montreal Cognitive Assessment (MoCA). Although these tools have demonstrated considerable clinical value, they are often time-consuming, require administration by trained professionals, and are not readily adaptable to continuous monitoring. Such limitations restrict their scalability and utility in large-scale cognitive screening programs, particularly in community- and home-based care settings (Wang R. et al., 2025). Consequently, the development of efficient, accessible, and sustainable intelligent screening approaches has become a major research priority in the prevention and management of cognitive disorders (Gutstein et al., 2026). Recent advances in artificial intelligence (AI) have accelerated the development of novel approaches for the early detection of AD. Traditional machine learning and deep learning methods have shown promise in language analysis, neuroimaging interpretation, and biomarker prediction. However, many existing models rely heavily on manual feature engineering or single-modality data, potentially limiting their generalizability and clinical applicability. Large language models (LLMs), characterized by their advanced capabilities in natural language understanding, complex reasoning, and multimodal information processing, can automatically extract cognitive features from diverse forms of unstructured data, including speech, text, and electronic health records. These capabilities position LLMs as promising tools for cognitive impairment detection, automated cognitive assessment, and clinical decision support in AD screening and care (AlSaad et al., 2024; Meng et al., 2024). Research exploring the use of LLMs for AD screening has expanded rapidly in recent years, accompanied by increasingly diverse application scenarios. However, substantial heterogeneity exists across studies regarding model types, data sources, methodological frameworks, and application strategies. To date, a comprehensive synthesis of this emerging body of evidence remains lacking. Therefore, this scoping review aims to systematically map the current evidence landscape, application domains, and evolving research directions of LLMs in the early detection of AD, thereby informing the future development of intelligent cognitive screening technologies and facilitating their integration into clinical and nursing practice.

## 2 Materials and methods

### 2.1 Review questions

This scoping review was conducted to address the following research questions:

1. What types of large language models (LLMs) have been applied in the early detection of Alzheimer’s disease (AD)?

2. What data sources have been used to support LLM-based AD screening?

3. What roles do LLMs play in the early detection and screening of AD?

4. What prompting strategies are adopted by LLMs in early AD screening?

### 2.2 Eligibility criteria

Studies were considered eligible if they met the following criteria: (1) participants included individuals with AD, mild cognitive impairment (MCI), or cognitively healthy controls; (2) LLMs were applied for the early screening, identification, detection, or risk prediction of cognitive impairment. In this study, LLMs were defined as artificial intelligence models based on large-scale pretrained models with capabilities in natural language understanding, generation, or language information processing. The included models comprised text-based large language models primarily designed for language processing, multimodal large language models capable of processing multiple information sources such as text, images, and speech, and medical-specific models based on LLM architectures with domain-specific language understanding or generation capabilities; (3) the study was an original research article employing quantitative, qualitative, or mixed-methods designs; and (4) the full text was accessible and provided sufficient methodological and outcome information. Studies were excluded if they were (1) review articles, commentaries, conference abstracts, dissertations, or duplicate publications; or (2) unavailable in full text or lacking sufficient information for data extraction and analysis.

### 2.3 Search strategy

The search strategy was developed in accordance with the Joanna Briggs Institute (JBI) methodology for scoping reviews. A comprehensive literature search was conducted in PubMed, Web of Science, Embase, Cochrane Library, ProQuest, and Scopus from database inception to April 20, 2026. All studies meeting the predefined inclusion criteria were considered eligible for screening. The search strategy combined controlled vocabulary terms and free-text keywords and was structured around three core concepts: (1) Alzheimer’s disease and mild cognitive impairment; (2) large language models and natural language processing; and (3) screening, detection, and prediction. Search terms included “Alzheimer’s disease,” “mild cognitive impairment,” “large language model,” “natural language processing,” “screening,” “detection,” and “prediction.” Terms within the same concept were combined using the Boolean operator “OR,” whereas concepts were combined using “AND.” The search strategy was adapted as appropriate for each database. The complete PubMed search strategy is provided in Supplementary Material 1. To ensure comprehensive retrieval of relevant evidence, the reference lists of all included studies were manually screened to identify additional eligible publications. Literature searching, screening, and deduplication were independently conducted by two reviewers. Any disagreements were resolved through discussion or consultation with a third reviewer.

### 2.4 Study selection and data extraction

Study selection and data extraction were independently conducted by two reviewers. All retrieved records were imported into NoteExpress software for deduplication. After duplicate records were removed, titles and abstracts were screened according to the eligibility criteria. Potentially relevant studies subsequently underwent full-text review to determine final inclusion. Disagreements between reviewers were resolved through discussion and, when necessary, adjudicated by a third reviewer. Data were extracted using a standardized charting form developed *a priori* and independently verified by both reviewers. Extracted information included the first author, country of origin, study design, assessment tools, study population and/or data sources, applications of LLMs, and principal findings. All stages of the literature screening process were independently performed by the researchers, and no artificial intelligence tools or large language models were used to assist with study selection.

### 2.5 Methodological considerations and quality assessment

This study followed the PRISMA-ScR (Preferred Reporting Items for Systematic Reviews and Meta-Analyses Extension for Scoping Reviews) guidelines and the methodological guidance provided by the JBI Evidence Synthesis Manual. According to the methodological framework of scoping reviews, the primary objective of this study was to map and describe the current evidence, key concepts, and research gaps regarding the application of LLMs in early AD screening, rather than to formally assess study quality or synthesize effect estimates. Therefore, no risk-of-bias assessment or methodological quality appraisal was conducted for the included studies. To ensure methodological rigor and transparency, this study applied independent procedures and cross-checking by two researchers throughout protocol registration (OSF registration number: 10.17605/OSF.IO/CXEFG), literature searching, screening, and data extraction. The section “3 Results” systematically described the characteristics of each included study, including study design, sample information, data sources, LLM types, and application approaches. This approach is consistent with the objectives of scoping reviews, which emphasize evidence mapping and characterization of research landscapes.

## 3 Results

### 3.1 Study selection

The study selection process was conducted in accordance with the Preferred Reporting Items for Systematic Reviews and Meta-Analyses Extension for Scoping Reviews (PRISMA-ScR) guidelines. A total of 3,458 records were identified through the literature search. After duplicate removal using the NoteExpress software, 2,212 records remained for title and abstract screening. Subsequently, the titles and abstracts were screened by the researchers, and 2,084 irrelevant records were excluded. A total of 128 articles underwent full-text screening. Finally, 103 articles were excluded, including 12 articles without available full texts, 29 non-original studies, 35 studies unrelated to cognitive screening, and 27 studies that did not involve the application of LLMs. Ultimately, 25 studies were included in this review. The literature screening process is shown in Figure 1.

**FIGURE 1:**
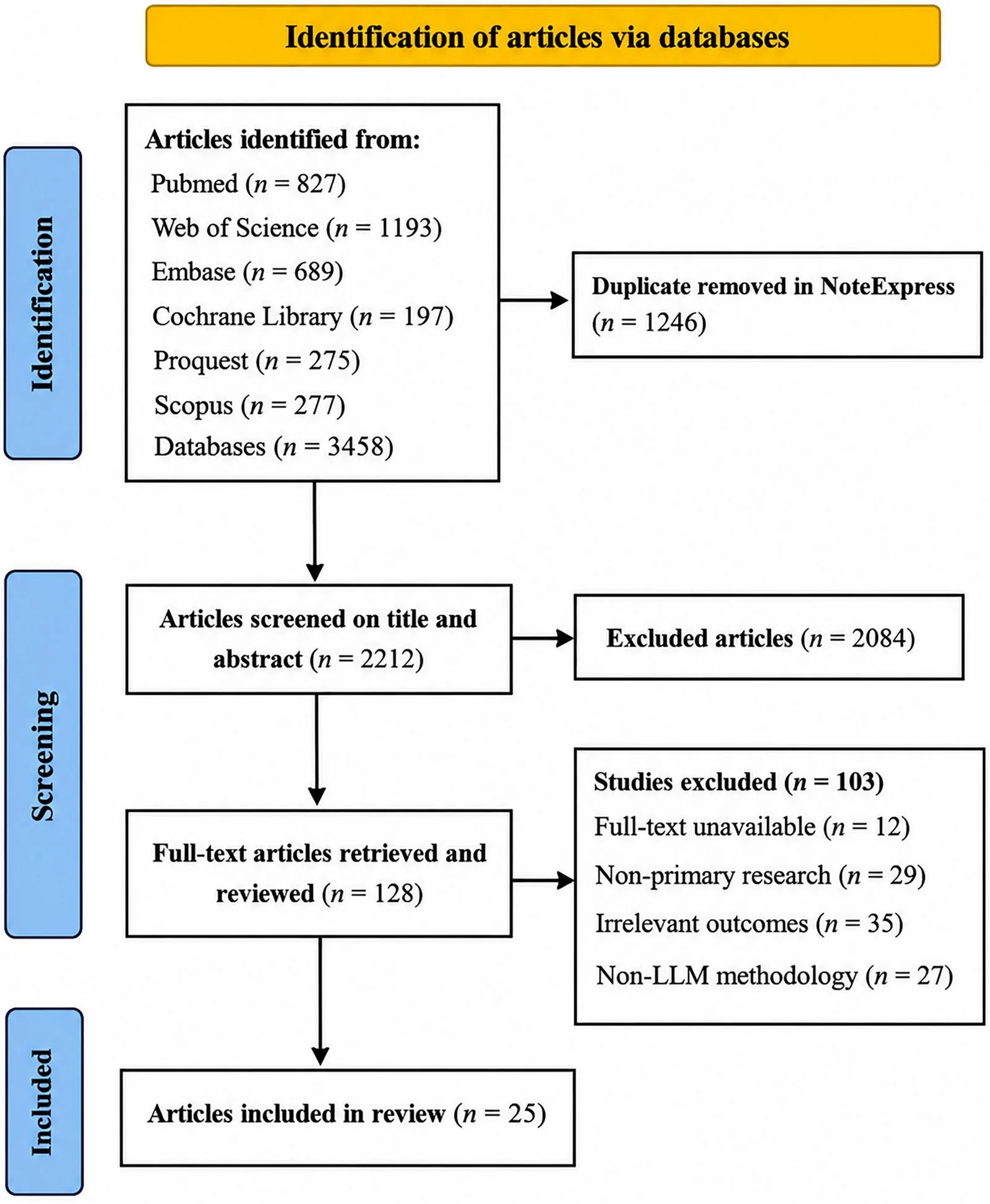
Flow diagram of literature search and screening.

### 3.2 Characteristics of included studies

The 25 included studies were published between 2022 and 2026. Most studies were published after 2024, indicating that the application of LLMs in early AD screening represents a rapidly emerging research field in recent years. The studies were conducted primarily in the United States (*n* = 9, 36%) (Agbavor and Liang, 2022; Guan et al., 2025; Huang et al., 2025; Kearney et al., 2025; Knox et al., 2025; Leng et al., 2025; Li et al., 2025; Zhan et al., 2025; Zolnour et al., 2025), followed by China (*n* = 6, 24%) (Han et al., 2025; Chen et al., 2026; Guo et al., 2025; Wang Y. et al., 2025; Zhang et al., 2025, 2026), South Korea (*n* = 3, 12%) (Bang et al., 2024; Cha et al., 2025; Park and Kim, 2025), Poland (*n* = 1, 4%) (Chlasta et al., 2025), Japan (*n* = 1, 4%) (Kiguchi et al., 2025), Singapore (*n* = 1, 4%) (Balamurali and Chen, 2024), Spain (*n* = 1, 4%) (Pérez-Elvira et al., 2026), Israel (*n* = 1, 4%) (Zadok et al., 2026), Italy (*n* = 1, 4%) (Casu et al., 2026), and Tunisia (*n* = 1, 4%) (Brahmi et al., 2024). All included studies employed quantitative research designs. Among them, 24 were observational studies (96%) (Agbavor and Liang, 2022; Balamurali and Chen, 2024; Bang et al., 2024; Brahmi et al., 2024; Casu et al., 2026; Cha et al., 2025; Chen et al., 2026; Chlasta et al., 2025; Guan et al., 2025; Guo et al., 2025; Han et al., 2025; Huang et al., 2025; Kearney et al., 2025; Kiguchi et al., 2025; Knox et al., 2025; Leng et al., 2025; Li et al., 2025; Park and Kim, 2025; Wang Y. et al., 2025; Zadok et al., 2026; Zhan et al., 2025; Zhang et al., 2025, 2026; Zolnour et al., 2025), and one was a computational simulation study (4%) (Pérez-Elvira et al., 2026). Overall, the existing literature has predominantly focused on model development and technical validation, whereas studies evaluating real-world clinical implementation remain limited. The characteristics of the included studies are presented in Table 1. Detailed information regarding dataset accessibility and Digital Object Identifier (DOI) information is provided in Supplementary Table 2.

**TABLE 1:** Characteristics of included studies.

No.	References	Country	Study design	Tools	Study population/ data source	Main role of LLMs	Main findings and evaluation metrics	LLM prompting strategies	Clinical translation-related considerations
1	Han et al., 2025	Hong Kong, China	Observational study	GPT-4, Llama 3.1	ADReSS dataset	Data augmentation	LLM-generated synthetic data improved MCI detection performance. GPT-4 combined with observational, cross-linguistic, and counterfactual generation strategies achieved the best performance, with a sensitivity of 42% and an F1-score of 35%.	No conventional prompting strategy reported; LLM generation strategy used for data augmentation	3
2	Park and Kim, 2025	South Korea	Observational study	Llama 3.2, Qwen2.5-VL	ADReSS dataset	Cognitive impairment identification	Chain-of-thought reasoning improved cognitive impairment detection performance and outperformed zero-shot, few-shot, and no-chain-of-thought baselines, achieving an accuracy of 86.67% ± 1.14% and an F1-score of 86.34% ± 0.85%.	Zero-shot prompting; few-shot prompting; chain-of-thought prompting	5
3	Huang et al., 2025	United States	Observational study	GPT-4o	Clinical data from five nursing home cohorts, fecal microbiome data, and knowledge base information	Cognitive impairment identification	The ADAM multi-agent framework integrating clinical and microbiome information outperformed conventional machine learning models, achieving a mean F1-score of 72.63%, higher than XGBoost (67.74%), with improved stability.	Chain-of-thought prompting; retrieval-augmented prompting; agent-based prompting	1
4	Guan et al., 2025	United States	Observational study	GatorTron, SCD-Tron	MGB healthcare system EHR text	Cognitive impairment identification	SCD-Tron achieved high performance in identifying subjective cognitive decline from clinical EHR text, with a precision of 88.12%, recall of 98.26%, F1-score of 92.89%, ROC-AUC of 99.93%, and PR-AUC of 98.04%.	No generative prompting; fine-tuned classification model	1
5	Chlasta et al., 2025	Poland	Observational study	GPT-4o	PROCESS dataset	Cognitive impairment identification	LLM-extracted features were used for cognitive decline classification and cognitive score prediction. The classification model achieved an accuracy of 58.7% and an F1-score of 55.2%, while the best regression model achieved an RMSE of 2.7775.	Conventional prompting strategy not clearly reported; LLM-based feature extraction	–
6	Zolnour et al., 2025	United States	Observational study	GPT-4o, MedAlpaca-7B	ADReSSo 2021, DementiaBank corpus	Data augmentation	LLM-generated synthetic speech data further improved cognitive impairment screening performance. With two-fold augmentation using MedAlpaca-7B, the F1-score reached 85.65% on the ADReSSo test set; one-fold augmentation achieved an F1-score of 72.82% and an AUC of 69.57% in an external MCI validation cohort.	Role prompting; LLM generation strategy	–
7	Kiguchi et al., 2025	Japan	Observational study	GPT-4o; Claude 3.5 Sonnet; Gemini 2.0	OASIS, ADNI, and publicly available AD-related EEG datasets	Data augmentation	LLMs combined with knowledge graphs enabled cross-modal integration and hypothesis generation from fragmented multimodal AD data. The consistency rate among three LLMs for major findings was 78%, with an expert-rated Cohen’s κ of 0.82.	Retrieval-augmented prompting	–
8	Balamurali and Chen, 2024	Singapore	Observational study	ChatGPT-3.5/4, Bard	ADReSSo dataset	Cognitive impairment identification	ChatGPT and Bard were able to distinguish AD patients from cognitively normal individuals under zero-shot conditions; however, overall performance remained insufficient for clinical application. Bard achieved a sensitivity of 89% and an F1-score of 71% for AD identification, while GPT-4 achieved an F1-score of 62% for identifying cognitively normal individuals.	Zero-shot prompting; chain-of-thought prompting	4
9	Guo et al., 2025	China	Observational study	GPT-3.5/4	ADReSSo dataset	Cognitive impairment identification	GPT-4 achieved good AD detection performance without fine-tuning under zero-shot conditions, with both accuracy and F1-score reaching 83.3%, and provided interpretable step-by-step reasoning.	Zero-shot prompting; few-shot prompting; chain-of-thought prompting	2,3
10	Wang Y. et al., 2025	China	Observational study	Qwen2	DementiaBank and clinical multimodal dialogue system	Automated assessment	The intelligent Web-MMSE system enabled multi-turn cognitive assessment, automated scoring, and AD risk classification. The MMSE score prediction model achieved an RMSE of 4.11, and the AD classification model achieved an accuracy of 89.89% and an F1-score of 88.88%.	Role prompting	1
11	Zhan et al., 2025	United States	Observational study	Llama3, Qwen2.5	ADNI database	Cognitive impairment identification	Fine-tuned LLMs integrating genetic, demographic, and clinical information effectively distinguished different cognitive states. After incorporating GDS information, Qwen2.5-7B achieved the best performance, with an accuracy of 97.92%.	No generative prompting; structured task instructions used for supervised fine-tuning	–
12	Pérez-Elvira et al., 2026	Spain	Simulation study	GPT-4o-mini	LLM-generated virtual elderly cohort	Data augmentation	GPT-4o-mini-generated virtual cohorts simulated cognitive decline trajectories from cognitively normal status to MCI and AD. Significant differences were observed in 8 of 13 cognitive domains, with an effect size of Cohen’s *d* = 4.71 for episodic memory deficits and *d* = 3.07 for narrative length.	Zero-shot prompting; few-shot prompting; role prompting	–
13	Li et al., 2025	United States	Observational study	LLaMA 3.1	VHA longitudinal EHR	Clinical decision support	The CARE-AD multi-agent framework utilized longitudinal EHR data for early AD risk prediction. At 10 years before formal diagnosis, the accuracy was 53%, which was higher than single-model approaches (26%–45%).	Chain-of-thought prompting; role prompting; agent-based prompting	1
14	Agbavor and Liang, 2022	United States	Observational study	GPT-3	ADReSSo public speech transcripts	Cognitive impairment identification	GPT-3 text embeddings were used for AD identification. The Babbage embedding combined with SVC achieved an accuracy of 80.3% and an F1-score of 82.9% on the test set, outperforming traditional acoustic feature-based methods.	No generative prompting; GPT-3 used for text embedding extraction	–
15	Chen et al., 2026	China	Observational study	HuatuoGPT, GPT-4o	ADNI and four other datasets plus local cohorts	Clinical decision support	HoloDx integrated LLMs/expert knowledge with multimodal clinical data to improve AD diagnosis. It achieved an accuracy of 91.92% and an AUC of 88.23% on the AIBL dataset and generally outperformed baseline models across multiple datasets.	Retrieval-augmented prompting	1
16	Zhang et al., 2026	China	Observational study	GPT-4	ADNI database	Clinical decision support	The self-learning and critical multi-agent framework effectively integrated multimodal information for CN/MCI/AD classification. The final framework achieved an F1-score of 88%, sensitivity of 88%, and precision of 90%, outperforming single-dimension agents.	Role prompting; retrieval-augmented prompting; agent-based prompting	1,2
17	Knox et al., 2025	United States	Observational study	Me-LLaMA, Mistral	De-identified EHR notes from the MUSC research repository	Cognitive impairment identification	LLMs based on clinical EHR text improved ADRD identification performance. Me-LLaMA achieved the best performance, with an AUC of 95.34% and an F1-score of 85.71%, outperforming the conventional CCW algorithm.	Zero-shot prompting	1
18	Zadok et al., 2026	Israel	Observational study	GPT-4o, LLaMA 3	Pitt corpus	Cognitive impairment identification	LLMs used clinically relevant linguistic cues to identify cognitive impairment more extensively than non-experts; however, misclassification remained frequent. Among dementia cases diagnosed clinically, the recognition rate was 60%, with false negatives accounting for 70% of errors, while MCI recognition was only 26%.	Zero-shot prompting	2,4
19	Leng et al., 2025	United States	Observational study	GPT-4o	MGB system EHR and memory clinic notes	Cognitive impairment identification	The GPT-4o framework enabled cognitive stage identification from EHR data and outperformed conventional features and embedding-based methods. The weighted Cohen’s κ was 0.95, Spearman correlation coefficient was 0.93, Baccianella MSE was 0.02, and the best κ for automated CDR scoring was 0.83.	Retrieval-augmented prompting	1,4
20	Casu et al., 2026	Italy	Observational study	Llama2, Mistral-7B	ADReSSo 2021	Cognitive impairment identification	The multimodal model integrating fine-tuned Mistral-7B language features with VGGish acoustic features achieved the best performance. Combined with SVC, it achieved an accuracy of 93.0%, an F1-score of 92.8%, and ROC-AUC of 97.9%, outperforming unimodal methods and previous baselines.	No generative prompting; LoRA fine-tuning and language feature extraction	–
21	Zhang et al., 2025	China	Observational study	Qwen-VL-Max; Qwen-Audio-Turbo	Multimodal MMSE data from the First Affiliated Hospital of Chongqing Medical University	Cognitive impairment identification	Decision trees constructed based on LLM-extracted features effectively distinguished AD from cognitively normal individuals, achieving an accuracy of 76% ± 3%, recall of 82% ± 6%, and F1-score of 76% ± 12%. After incorporating counterfactual explanations, the best F1-score reached 78%.	Zero-shot prompting; role prompting	2
22	Cha et al., 2025	South Korea	Observational study	LLaMA 3.3, Phi-4	Hanyang University Hospital cohort	Clinical decision support	CLONE simulated neuropsychological experts and generated step-by-step diagnostic guidelines to improve MCI identification and reasoning performance. LLaMA 3.3 achieved the best performance, with an accuracy of 89.23% and an F1-score of 90.91%, representing a 6.15 percentage-point improvement over the best few-shot chain-of-thought baseline.	Zero-shot prompting; few-shot prompting; chain-of-thought prompting; role prompting; agent-based prompting	1
23	Brahmi et al., 2024	Tunisia	Observational study	LLaVA-Med	ADNI, OASIS, and AB-MRI datasets	Cognitive impairment identification	LLaVA-Med enabled AD classification using MRI images and outperformed conventional deep learning methods across three datasets. The best performance was achieved on the ADNI dataset, with an accuracy of 95.20% and an F1-score of 93.15%.	No explicit prompting strategy reported; LLaVA-Med fine-tuning	–
24	Kearney et al., 2025	United States	Observational study	Qwen2.5, GPT-4.1-mini	ADNI QT-PAD biomarker dataset	Cognitive impairment identification	TAP-GPT utilized multimodal biomarker tables for AD prediction. Under few-shot tabular prompting, it achieved the best performance, with an average F1-score of 80.95% ± 7.75% and an accuracy of 88.80% ± 4.84%, outperforming TabPFN and multiple general-purpose LLMs.	Zero-shot prompting; few-shot prompting; chain-of-thought prompting	2
25	Bang et al., 2024	South Korea	Observational study	GPT-4, GPT-3.5	237 recorded speech samples from ADReSSo	Cognitive impairment identification	Integrating ChatGPT-generated linguistic evaluation comments with speech and text information improved AD recognition performance. The best multimodal model achieved an accuracy of 87.32%, F-score of 87.25%, and specificity of 94.44%.	Few-shot prompting	–

Percentage-based evaluation metrics were uniformly presented as percentages, whereas other metrics were reported according to the scales used in the original studies. AD, Alzheimer’s disease; MCI, mild cognitive impairment; CN, cognitively normal; LLMs, large language models; ADRD, Alzheimer’s disease and related dementias; ADReSS, Alzheimer’s Dementia Recognition through Spontaneous Speech; ADReSSo, Alzheimer’s Dementia Recognition through Spontaneous Speech Challenge; ADNI, Alzheimer’s Disease Neuroimaging Initiative; OASIS, Open Access Series of Imaging Studies; MMSE, Mini-Mental State Examination; EHR, electronic health records; EEG, electroencephalography; GDS, Geriatric Depression Scale; SVC, support vector classifier; CCW, Chronic Conditions Warehouse; AUC, area under the curve; ROC-AUC, area under the receiver operating characteristic curve; PR-AUC, area under the precision–recall curve; F1, F1 score; RMSE, root mean square error; MSE, mean squared error; LoRA, Low-Rank Adaptation. Clinical translation-related considerations were coded as follows: 1, clinical application/workflow exploration; 2, interpretability/explainability; 3, bias/fairness; 4, safety/potential clinical risk; and 5, privacy/data protection. “–” indicates that none of the above considerations was explicitly reported in the original study. A category was coded only when the corresponding issue was explicitly reported, evaluated, or discussed in the original study. The categories were non-mutually exclusive, and more than one code could be assigned to the same study.

### 3.3 Types of LLMs applied in early AD screening

The LLMs used in the included studies were classified into three categories: general-purpose generative LLMs, multimodal LLMs, and medical-specific LLMs. (1) General-purpose generative LLMs were applied in 17 studies (68%) (Agbavor and Liang, 2022; Balamurali and Chen, 2024; Bang et al., 2024; Casu et al., 2026; Cha et al., 2025; Chen et al., 2026; Guo et al., 2025; Han et al., 2025; Kearney et al., 2025; Kiguchi et al., 2025; Knox et al., 2025; Li et al., 2025; Pérez-Elvira et al., 2026; Wang Y. et al., 2025; Zadok et al., 2026; Zhan et al., 2025; Zhang et al., 2026), including GPT, LLaMA, Qwen, Mistral, Phi, Bard, and Claude. These models were mainly used for text understanding, cognitive status identification, and clinical reasoning tasks. (2) Multimodal LLMs were used in 9 studies (36%) (Brahmi et al., 2024; Chlasta et al., 2025; Huang et al., 2025; Kiguchi et al., 2025; Leng et al., 2025; Park and Kim, 2025; Zadok et al., 2026; Zhang et al., 2025; Zolnour et al., 2025), including GPT-4o, the Gemini series, Qwen-VL, and LLaVA. These models are capable of simultaneously processing multiple types of information, such as text, speech, and images, enabling cross-modal feature extraction and integrated analysis. (3) Medical-specific LLMs were applied in 3 studies (12%) (Guan et al., 2025; Knox et al., 2025; Zolnour et al., 2025), including GatorTron, SCD-Tron, MedAlpaca, and Me-LLaMA. These models were pretrained or fine-tuned on medical corpora and are more suitable for professional knowledge understanding and reasoning in healthcare scenarios. Some studies employed multiple types of LLMs for model comparison or collaborative analysis; therefore, overlap exists among the different model categories. Overall, general-purpose generative LLMs remain the predominant models used in current AD early screening studies. However, as the complex phenotypic characteristics of AD and the need for multidimensional clinical assessment become increasingly recognized, multimodal LLMs are receiving growing attention. Future integration of diverse medical information may represent an important direction for improving the clinical applicability and translational potential of LLMs in AD early screening.

### 3.4 Data sources for LLM-based AD early detection

The data sources used in the included studies were classified into three categories: publicly available datasets, real-world clinical data, and synthetic data. A total of 17 studies (68%) (Agbavor and Liang, 2022; Balamurali and Chen, 2024; Bang et al., 2024; Brahmi et al., 2024; Casu et al., 2026; Chen et al., 2026;

Chlasta et al., 2025; Guo et al., 2025; Han et al., 2025; Kearney et al., 2025; Kiguchi et al., 2025; Park and Kim, 2025; Wang Y. et al., 2025; Zadok et al., 2026; Zhan et al., 2025; Zhang et al., 2026; Zolnour et al., 2025) utilized publicly available datasets. Commonly used resources included ADReSS, ADReSSo, DementiaBank, Pitt corpus, ADNI, OASIS, and PROCESS datasets. Speech- and text-based datasets were the most frequently used and primarily supported the extraction of linguistic and cognitive features. In addition, multimodal datasets provided neuroimaging, electroencephalography (EEG), biomarker, and cognitive assessment data, enabling the development of multimodal screening models. Seven studies (28%) (Cha et al., 2025; Guan et al., 2025; Huang et al., 2025; Knox et al., 2025; Leng et al., 2025; Li et al., 2025; Zhang et al., 2025) employed real-world clinical data, including electronic health records, clinical notes, follow-up data from long-term care facilities, and clinical cohort datasets. These data sources more closely reflect disease trajectories in routine clinical practice and facilitate evaluation of model performance in real-world healthcare settings. One study (4%) (Pérez-Elvira et al., 2026) utilized synthetic data generated by LLMs, including virtual cohorts of older adults and simulated conversational data, to support model development and validation. This approach was primarily used to address challenges associated with limited sample sizes and restricted data availability. Publicly available datasets constituted the predominant data source across the included studies, whereas investigations based on real-world clinical data remained relatively limited.

### 3.5 Roles of LLMs in early AD screening

The included studies demonstrated that the roles of LLMs in early AD screening mainly involved four aspects: cognitive impairment identification, data augmentation, automated assessment, and clinical decision support. (1) Cognitive impairment identification was reported in 16 studies (64%) (Agbavor and Liang, 2022; Balamurali and Chen, 2024; Bang et al., 2024; Brahmi et al., 2024; Casu et al., 2026; Chlasta et al., 2025; Guan et al., 2025; Guo et al., 2025; Huang et al., 2025; Kearney et al., 2025; Knox et al., 2025; Leng et al., 2025; Park and Kim, 2025; Zadok et al., 2026; Zhan et al., 2025; Zhang et al., 2025). By analyzing speech transcripts, clinical records, and natural conversation data, LLMs extracted language features and cognitive-related information to achieve the identification and classification of AD and mild cognitive impairment. (2) Data augmentation was investigated in 4 studies (16%) (Han et al., 2025; Kiguchi et al., 2025; Pérez-Elvira et al., 2026; Zolnour et al., 2025). These studies utilized LLMs to generate or expand synthetic data, increase training sample sizes, alleviate data imbalance, and improve overall model performance. (3) Automated assessment was reported in 1 study (4%) (Wang Y. et al., 2025). This study developed an automated cognitive assessment system that enabled preliminary screening through intelligent dialogue guidance, cognitive scale prediction, and real-time cognitive status assessment. (4) Clinical decision support was explored in 4 studies (16%) (Cha et al., 2025; Chen et al., 2026; Li et al., 2025; Zhang et al., 2026). LLMs were applied to simulate multidisciplinary expert consultation, disease reasoning, and potential biomarker analysis, providing auxiliary evidence for the diagnosis of cognitive disorders.

### 3.6 Prompting strategies used in LLM-based early AD screening

The prompting strategies applied in the included studies mainly included zero-shot prompting, few-shot prompting, chain-of-thought prompting, role prompting, retrieval-augmented prompting, and agent-based prompting. (1) Zero-shot prompting was adopted in 9 studies (36%) (Balamurali and Chen, 2024; Cha et al., 2025; Guo et al., 2025; Kearney et al., 2025; Knox et al., 2025; Park and Kim, 2025; Pérez-Elvira et al., 2026; Zadok et al., 2026; Zhang et al., 2025). This strategy primarily involved directly providing task instructions to LLMs to perform cognitive status identification and classification tasks. (2) Few-shot prompting was used in 6 studies (24%) (Bang et al., 2024; Cha et al., 2025; Guo et al., 2025; Kearney et al., 2025; Park and Kim, 2025; Pérez-Elvira et al., 2026). By providing a small number of examples, this strategy assisted models in understanding task requirements and adapting to specific tasks. (3) Chain-of-thought prompting was applied in 7 studies (28%) (Balamurali and Chen, 2024; Cha et al., 2025; Guo et al., 2025; Huang et al., 2025; Kearney et al., 2025; Li et al., 2025; Park and Kim, 2025). This strategy was mainly used to facilitate step-by-step analysis and complex reasoning processes. (4) Role prompting was adopted in 7 studies (28%) (Cha et al., 2025; Li et al., 2025; Pérez-Elvira et al., 2026; Wang Y. et al., 2025; Zhang et al., 2025, 2026; Zolnour et al., 2025). By assigning specific task roles to LLMs, this strategy aimed to enhance model adaptability. (5) Retrieval-augmented prompting was reported in 5 studies (20%) (Chen et al., 2026; Huang et al., 2025; Kiguchi et al., 2025; Leng et al., 2025; Zhang et al., 2026). This strategy incorporated external knowledge to assist model analysis. (6) Agent-based prompting was used in 4 studies (16%) (Cha et al., 2025; Huang et al., 2025; Li et al., 2025; Zhang et al., 2026). Through multi-agent collaboration, these approaches enabled information integration and reasoning tasks. Some studies combined multiple prompting strategies in task design, such as integrating chain-of-thought prompting, role prompting, retrieval augmentation, or agent-based strategies, to address complex cognitive screening tasks. In addition, 6 studies (24%) (Agbavor and Liang, 2022; Brahmi et al., 2024; Casu et al., 2026; Chlasta et al., 2025; Guan et al., 2025; Zhan et al., 2025) did not employ conventional prompting strategies but instead used model fine-tuning, language feature extraction, text embedding, or other task-adaptation approaches to perform cognitive screening tasks. Another 2 studies (8%) (Han et al., 2025; Zolnour et al., 2025) utilized LLM-generated synthetic data augmentation to improve model performance. Furthermore, some studies suggested that optimization of prompting strategies may be associated with improved model performance. Park and Kim (2025) found that introducing chain-of-thought prompting improved cognitive impairment detection performance compared with zero-shot, few-shot, and non-chain-of-thought baselines, achieving an accuracy of 86.67% and an F1 score of 86.34%. Cha et al. (2025) developed the CLONE framework, which integrated role prompting, chain-of-thought reasoning, and agent-based collaborative reasoning. The framework achieved an F1 score of 90.91%, representing a 6.15 percentage-point improvement compared with the best few-shot chain-of-thought baseline. Huang et al. (2025) and Zhang et al. (2026) further demonstrated that combining retrieval augmentation with agent-based strategies could facilitate multi-source information integration and improve reasoning performance in complex cognitive tasks. However, due to differences in data sources, task types, and evaluation metrics across studies, it remains unclear whether any specific prompting strategy has universal superiority.

### 3.7 Clinical translation-related considerations in LLM-based early AD screening

Clinical translation-related considerations identified in the included studies mainly involved five dimensions: clinical application/workflow exploration, interpretability/explainability, bias/fairness, safety/potential clinical risk, and privacy/data protection. (1) Clinical application or workflow exploration was reported in 9 studies (36%) (Cha et al., 2025; Chen et al., 2026; Guan et al., 2025; Huang et al., 2025; Knox et al., 2025; Leng et al., 2025; Li et al., 2025; Wang Y. et al., 2025; Zhang et al., 2026), mainly involving cognitive status identification using clinical data, automated cognitive assessment, electronic health record analysis, risk prediction, and clinical decision support. (2) Interpretability or explanation of model outputs was addressed in 5 studies (20%) (Guo et al., 2025; Kearney et al., 2025; Zadok et al., 2026; Zhang et al., 2025, 2026), mainly through step-by-step reasoning, analysis of the basis for model judgments, counterfactual explanations, and interpretable feature extraction. (3) Bias- or fairness-related issues were considered in 2 studies (8%) (Guo et al., 2025; Han et al., 2025), primarily involving differences in data distributions, sample representativeness, and model generalizability across populations. (4) Model safety or potential clinical risks were addressed in 3 studies (12%) (Balamurali and Chen, 2024; Leng et al., 2025; Zadok et al., 2026), including prediction reliability, discrepancies between human and model judgments, and misclassification risks. (5) Privacy- or data-protection-related issues were reported in 1 study (4%) (Park and Kim, 2025). In addition, no included study systematically evaluated regulatory requirements or governance frameworks for the application of LLMs in early AD screening (0%). These five dimensions were non-mutually exclusive, and individual studies could be assigned to more than one dimension; the corresponding codes are presented in Table 1. The use of real-world clinical data has been described in the preceding Data Sources section.

## 4 Discussion

### 4.1 LLMs promote the transition of early AD screening from manual feature extraction to semantic intelligent analysis

Among the 25 studies included in this review, 17 utilized speech transcripts, clinical records, or natural conversational data to identify cognitive abnormalities, indicating that language information analysis has become an important application direction of LLMs in early AD screening. Language impairment represents an early manifestation of AD, with patients potentially exhibiting word-finding difficulties, abnormal semantic expression, and reduced narrative coherence, which may occur before prominent memory impairment and functional decline (Marier et al., 2026). Therefore, language-based cognitive screening provides advantages including non-invasiveness, convenience, and repeatable assessment, offering an important foundation for applying LLMs to the identification of early cognitive abnormalities. However, it should be noted that language impairment is not specific to AD. Similar alterations, including reduced verbal expression, impaired lexical retrieval, and decreased narrative ability, may also occur in depression, vascular cognitive impairment, frontotemporal dementia, and normal aging (Geraudie et al., 2021; Viduani et al., 2026; Macoir, 2024; Shafto and Tyler, 2014). In addition, some patients with early AD may primarily present with impairments in memory, visuospatial abilities, or executive function while maintaining relatively preserved language abilities (Dickerson et al., 2017). Therefore, although language information can reflect certain cognitive changes, it cannot fully represent the clinical phenotype of AD or adequately distinguish different cognitive disorders. Consequently, LLM-based language analysis should be considered an auxiliary approach for early AD screening rather than an independent diagnostic method, and its clinical value depends on integration with multidimensional evidence, including neuropsychological assessments, neuroimaging, and biomarkers.

Compared with traditional machine learning approaches that rely on manually engineered linguistic features, such as word frequency, syntactic complexity, and pause patterns, LLMs acquire language representations through large-scale pretraining and can capture deeper semantic information and cognition-related features from unstructured text, facilitating a transition from handcrafted feature extraction toward semantic-based intelligent analysis in AD screening (Shankar et al., 2025). The included studies demonstrated that various LLMs, including GPT, LLaMA, and Qwen, have been applied to AD and mild cognitive impairment identification tasks, with different model architectures showing distinct application characteristics. Closed-source models represented by the GPT series generally exhibit strong general language understanding and reasoning capabilities, enabling various language tasks under zero-shot or few-shot settings. In contrast, open-source models such as LLaMA and Qwen provide advantages in accessibility, fine-tuning flexibility, and deployment adaptability, making them suitable for optimization using domain-specific data (Kumar, 2024; Minaee et al., 2024).

It should be further emphasized that the development of LLMs does not imply the replacement of traditional machine learning and deep learning approaches in early AD screening, but rather represents a further extension of artificial intelligence methods in terms of information processing capabilities and task adaptability. Traditional machine learning and deep learning models remain valuable for structured clinical indicator analysis, risk prediction, imaging feature extraction, and tasks requiring explicit interpretation of predefined variables (Prinzi et al., 2024). However, these approaches often rely on manually designed features when processing unstructured language information, limiting their ability to capture complex semantic relationships and latent cognitive representations. In contrast, LLMs acquire enhanced capabilities in natural language understanding, contextual modeling, and semantic reasoning through large-scale pretraining, enabling the extraction of high-dimensional semantic features from complex linguistic data, including electronic health records, speech transcripts, and natural conversations. Therefore, the value of LLMs in early AD screening lies not in replacing existing AI technologies, but in providing complementary capabilities for language understanding, knowledge integration, and unstructured data processing.

Furthermore, current studies demonstrate a transition in data sources from structured cognitive tasks toward more naturalistic language scenarios. Beyond traditional cognitive assessment transcripts, emerging studies have begun to incorporate information from image descriptions, spontaneous speech, and natural conversations for cognitive status analysis (Qi et al., 2023). This trend indicates that LLM applications are expanding beyond fixed-task disease classification toward the exploration of potential cognitive change patterns embedded in natural language behaviors. With further improvements in long-context understanding, multi-turn interaction, and reasoning capabilities, LLMs may support continuous cognitive state monitoring in the future, providing new opportunities for shifting AD early screening from episodic assessment toward dynamic evaluation.

### 4.2 Multimodal integration and agent-based collaborative reasoning facilitate the development of comprehensive cognitive assessment in early AD screening

Our review indicates that research on LLMs for early AD screening is gradually shifting from single-modality language analysis toward multimodal data integration and collaborative reasoning. This trend not only reflects advances in the technical capabilities of LLMs but also corresponds to the clinical reality that AD is characterized by multidimensional and multilevel pathological changes. Unlike purely language-based tasks, AD identification and assessment rely on multiple sources of evidence, including cognitive performance, neuroimaging, biomarkers, and clinical information. Therefore, LLM-based screening approaches inherently face limitations when relying solely on a single modality. Among the included studies, several have combined different data types, including speech, text, brain MRI, EEG, biomarkers, and electronic health records, with LLMs to integrate disease-related information and achieve more comprehensive cognitive assessment (Chen et al., 2026; Kiguchi et al., 2025). Different types of data provide complementary information for early AD screening. Language and textual information can reflect patients’ expressive abilities, semantic organization, and communication patterns, whereas neuroimaging, EEG, and biomarkers provide insights into structural and functional alterations of the brain. The development of multimodal LLMs has further facilitated the joint analysis of heterogeneous data sources, enabling models to simultaneously process text, speech, and imaging information, thereby providing new approaches for identifying complex cognitive change patterns. Compared with single-source data analysis, multimodal integration better reflects the multifactorial nature of AD progression. For LLMs, multimodal integration is not merely an optimization strategy for improving model performance, but also an essential foundation for overcoming the limitations of language-based analysis, enhancing disease representation, and facilitating future clinical translation. Given that AD involves abnormalities across multiple levels, including cognitive function, neural structure, and biological processes, future clinical applications of LLMs will require further integration of language information with imaging, biomarkers, and other medical data to enable more comprehensive evaluation of cognitive status.

Beyond data integration, agent-based collaborative reasoning has emerged as an emerging direction in the application of LLMs for AD screening in recent years (Mehandru et al., 2024). Some studies have developed agent-based collaborative frameworks in which different agents perform specific tasks, such as information extraction, knowledge retrieval, and result interpretation. This approach enables multi-step reasoning during complex cognitive assessment processes and extends LLM applications beyond direct classification tasks toward more comprehensive information integration and reasoning-based analysis (Zhang et al., 2026).

Overall, current applications of LLMs in early AD screening are gradually evolving from single-modality language analysis toward multimodal information integration and from single-model prediction toward collaborative reasoning frameworks. Future advances in multimodal data integration and model reasoning capabilities may not only improve the comprehensiveness of cognitive assessment but also facilitate the translation of LLM-based approaches from experimental research settings into real-world clinical practice.

### 4.3 Current challenges in the clinical translation of LLMs

#### 4.3.1 Limited generalizability in real-world clinical settings

Although LLMs have demonstrated considerable potential in early AD screening, current studies remain predominantly focused on model development and performance evaluation, with a considerable gap between research validation and real-world clinical implementation. In this study, most included studies were conducted using publicly available datasets, such as ADReSS, ADReSSo, DementiaBank, and ADNI. These datasets provide advantages including standardized data collection and well-defined labels, facilitating model training, performance comparison, and methodological validation. However, differences in data acquisition environments and patient characteristics compared with real clinical settings may limit the generalizability of LLMs across diverse populations and healthcare environments (Marconi and Cabitza, 2025). For example, publicly available speech and text datasets usually involve relatively standardized task designs and data collection procedures, whereas patients in real-world clinical settings exhibit greater heterogeneity in age, educational background, linguistic and cultural context, disease stage, and communication patterns. When models are applied to new environments with distributions different from the training data, distribution shifts may occur, potentially resulting in decreased model performance and reduced predictive stability (Koch et al., 2024). Therefore, future studies should prioritize external validation across multiple centers, populations, and linguistic environments to improve the reliability and transferability of LLM-based approaches for real-world AD screening.

#### 4.3.2 Challenges in multimodal data integration and standardization

In addition, current applications of LLMs in AD screening remain limited by insufficient data dimensionality. Most existing studies primarily focus on language or textual information, whereas clinical assessment of AD generally requires the integration of multiple sources of information, including cognitive assessments, neuroimaging, biomarkers, and disease-related clinical characteristics. Although language features can reflect alterations in patients’ expressive ability, semantic organization, and cognitive changes, a single language modality may not fully capture the complex pathological processes of AD and may increase uncertainty in distinguishing AD from other cognitive disorders (Dhareshwar et al., 2024). Therefore, successful clinical translation of LLMs requires not only the further development of multimodal fusion approaches but also the establishment of standardized frameworks for data acquisition, integration, and validation. However, real-world multimodal data remain challenged by issues related to data quality, privacy protection, cross-institutional data sharing, and data standardization, which further complicate the clinical implementation of LLM-based systems.

#### 4.3.3 Limited evidence for clinical workflow integration and responsible deployment

The clinical value of LLMs depends not only on model performance but also on their effective integration into healthcare workflows and their ability to meet key requirements for interpretability, fairness, safety, privacy protection, and regulatory governance (Wang and Zhang, 2024). The findings of this review indicate that current studies have begun to explore the use of LLMs in clinically oriented scenarios, including cognitive risk screening, automated cognitive assessment, electronic health record analysis, and clinical decision support, suggesting a gradual shift from isolated performance evaluation toward clinical application. However, clinical implementation involves more than applying a model to a specific healthcare task; it also requires clarification of the model’s role in information acquisition, result presentation, clinician review, and final decision-making, as well as evaluation of whether its use actually improves workflow efficiency and decision quality. With respect to interpretability, existing studies have explored approaches such as step-by-step reasoning, counterfactual explanations, and interpretable feature extraction to improve the understandability of model outputs. Nevertheless, the ability to generate an explanation does not necessarily mean that the explanation faithfully reflects the basis of model predictions, and further validation is needed to determine its reliability, stability, and practical value for clinical decision-making. Bias and fairness are also important considerations because AD screening may be influenced by age, educational level, language, and cultural background, and insufficient representation of diverse populations in training data may lead to heterogeneous model performance across patient groups. Future studies should therefore strengthen subgroup analyses and external validation in more diverse populations. From a safety perspective, evaluation should extend beyond overall predictive performance to include misclassification, model uncertainty, discrepancies between human and model judgments, and other abnormal outputs, together with mechanisms for clinician oversight, error feedback, and continuous risk monitoring. In addition, as LLMs are increasingly applied to electronic health records, speech, imaging, and other sensitive health information, privacy protection, data access control, and cross-institutional data sharing will become increasingly important. The absence of systematic evaluation of regulatory or governance frameworks among the included studies further highlights an important gap in the current evidence base for clinical translation. Future research should therefore extend beyond technical performance to address accountability, continuous monitoring, ethical oversight, and appropriate regulatory governance for medical LLM applications.

Overall, LLM-based approaches for early AD screening are currently transitioning from technical validation toward clinical translation. LLMs acquire language pattern recognition and information generation capabilities through large-scale training data; however, their outputs do not necessarily represent clinical reasoning in the medical sense. When patients present characteristics beyond the model training distribution or involve complex, ambiguous, or borderline cases, LLMs may generate incorrect predictions or uncertain outputs. Therefore, the appropriate role of LLMs in early AD screening should be as supportive tools for information analysis and clinical decision assistance rather than replacements for independent clinical diagnosis by physicians. Future research should focus on conducting multicenter real-world studies, advancing multimodal information integration, improving clinical workflow adaptation, and establishing comprehensive evaluation and regulatory frameworks to enhance the safety, reliability, and scalability of LLM-based applications in AD early screening.

### 4.4 Limitations

This study has several limitations. First, only studies published in English were included, while studies published in other languages were not considered, which may have introduced language bias. In addition, gray literature, including conference abstracts and dissertations, was excluded, potentially resulting in the omission of some exploratory research findings that have not yet been formally published. Second, the literature search in this study was conducted up to April 20, 2026. Given the rapid development of LLM technologies, newly published studies after this date may not have been captured. Therefore, the findings of this review primarily reflect the current state of LLM applications in early AD screening before this time point. Third, as a scoping review, this study followed the JBI methodological framework for evidence synthesis and focused on comprehensively mapping the application characteristics, research trends, and evidence distribution of LLMs in early AD screening. Methodological quality assessment of the included studies was not conducted; therefore, the overall quality of the available evidence could not be systematically determined. Furthermore, substantial heterogeneity existed among the included studies in terms of model types, data sources, study designs, and evaluation metrics, which may limit direct comparisons across studies. In addition, because current research provides relatively limited reporting regarding the clinical implementation process, real-world deployment, and related safety governance issues of LLMs, this review was unable to further evaluate their long-term effectiveness in actual healthcare environments. Future research should expand the scope of investigation, conduct more high-quality real-world studies, and integrate systematic review approaches to further strengthen the evidence base in this emerging field.

## 5 Conclusion

This study employed a scoping review methodology to systematically summarize the current applications of LLMs in early AD screening. Existing evidence indicates that LLMs have been applied in various areas, including cognitive impairment identification, data augmentation, automated cognitive assessment, and clinical decision support, with language-based cognitive impairment identification remaining the primary research direction. With the advancement of model capabilities, multimodal integration and agent-based collaborative reasoning have gradually emerged as important directions for future development. However, current research remains predominantly based on publicly available datasets, and further improvements are needed in real-world validation, multicenter studies, model generalizability, and ethical governance. Future research should strengthen investigations in clinical application scenarios and promote the standardized implementation of LLMs in AD risk identification, early assessment, and intelligent nursing practice.
