# Supplementary material for: Application of large language models in early screening of Alzheimer’s disease: a scoping review
Source: Front Aging Neurosci. 2026 Oct 12;18:1906641. doi: 10.3389/fnagi.2026.1906641 (PMC13697241; doi:10.3389/fnagi.2026.1906641)
Supplement: Supplementary file 1 [file Table_1.docx]

Supplementary Material S1

Database: PubMed

Date of search: April 20, 2026

Search conducted according to JBI methodological framework.

(("Alzheimer Disease"[Mesh] OR "Alzheimer*"[Title/Abstract] OR "mild cognitive impairment"[Title/Abstract] OR "cognitive impairment"[Title/Abstract]) AND ("large language model*"[Title/Abstract] OR LLM*[Title/Abstract]OR ChatGPT[Title/Abstract] OR GPT[Title/Abstract] OR "generative AI"[Title/Abstract] OR "natural language processing"[Mesh] OR "natural language processing"[Title/Abstract]) AND (screening[Title/Abstract] OR detection[Title/Abstract] OR identification[Title/Abstract] OR prediction[Title/Abstract] OR assessment[Title/Abstract]))

Search fields used: [Title/Abstract] and MeSH Terms

Time frame: From database inception to April 20, 2026

Other databases searched: Web of Science, Embase, Cochrane Library, ProQuest, and Scopus.

Additional step: Manual screening of reference lists of all included studies and relevant reviews.
