# Supplementary material for: Application of large language models in early screening of Alzheimer’s disease: a scoping review
Source: Front Aging Neurosci. 2026 Oct 12;18:1906641. doi: 10.3389/fnagi.2026.1906641 (PMC13697241; doi:10.3389/fnagi.2026.1906641)
Supplement: Supplementary file 2 [file Table_2.docx]

Supplementary Material S2

**Supplementary Table S2. Characteristics of included studies and dataset information**

| Study title | Reference number | Study design | LLMs type | Study population/data source | Data modality | Dataset accessibility | DOI |
| --- | --- | --- | --- | --- | --- | --- | --- |
| A large language model based data generation framework to improve mild cognitive impairment detection sensitivity | [8] | Observational study | GPT-4, Llama 3.1 | ADReSS dataset | Speech and text | Public | <https://doi.org/10.1017/dap.2025.8> |
| A novel chain-of-thought reasoning approach for Alzheimer’s disease detection using large language and vision-language models | [9] | Observational study | Llama 3.2, Qwen2.5-VL | ADReSS dataset | Speech and text | Public | <https://doi.org/10.1109/TNSRE.2025.3624762> |
| ADAM-1: an AI reasoning and bioinformatics model for Alzheimer’s disease detection and microbiome-clinical data integration | [10] | Observational study | GPT-4o | Clinical data from five nursing home cohorts, fecal microbiome data, and knowledge base information | Clinical data, microbiome data, and knowledge base information | Restricted | <https://doi.org/10.1109/ACCESS.2025.3599857> |
| CD-tron: leveraging large clinical language model for early detection of cognitive decline from electronic health records | [11] | Observational study | GatorTron, SCD-Tron | MGB healthcare system EHR text | Electronic health record text | Restricted | <https://doi.org/10.1016/j.jbi.2025.104830> |
| Enhancing dementia and cognitive decline detection with large language models and speech representation learning | [12] | Observational study | GPT-4o | PROCESS dataset | Speech and text | Public | <https://doi.org/10.3389/fninf.2025.1679664> |
| LLMCARE: early detection of cognitive impairment via transformer models enhanced by LLM-generated synthetic data | [13] | Observational study | GPT-4o, MedAlpaca | ADReSSo 2021, DementiaBank corpus | Speech and text | Public | <https://doi.org/10.3389/frai.2025.1669896> |
| Multi-modal integration analysis of Alzheimer’s disease using large language models and knowledge graphs | [14] | Observational study | GPT-4o；Claude 3.5 Sonnet；Gemini 2.0 | OASIS, ADNI, and publicly available AD-related EEG datasets | MRI, EEG, clinical data, and biomarkers | Public | <https://doi.org/10.1109/ACCESS.2025.3582853> |
| Performance assessment of ChatGPT versus Bard in detecting Alzheimer’s dementia | [15] | Observational study | ChatGPT-3.5/4、Bard | ADReSSo dataset | Speech and text | Public | <https://doi.org/10.3390/diagnostics14080817> |
| Interpretable Alzheimer’s disease detection with minimal data: zero-shot and few-shot approaches using large language models | [16] | Observational study | GPT-3.5/4 | ADReSSo dataset | Speech and text | Public | <https://doi.org/10.1007/978-981-96-6960-8_19> |
| An intelligent web-based interactive MMSE assessment system for Alzheimer’s disease using large language models | [17] | Observational study | Qwen2 | DementiaBank and clinical multimodal dialogue system | Speech, text, and cognitive assessment data | Public and Restricted | <https://doi.org/10.1109/CyberSciTech68397.2025.00097> |
| A general paradigm for fine-tuning large language models in Alzheimer’s disease diagnosis | [18] | Observational study | Llama3, Qwen2.5 | ADNI database | Imaging, genetic, demographic, and clinical data | Public | <https://doi.org/10.1609/aaaiss.v5i1.35550> |
| Architectural constraints in LLM-simulated cognitive decline: in silico dissociation of memory deficits and generative language as candidate digital biomarkers | [19] | Simulation study | GPT-4o-mini | LLM-generated virtual elderly cohort | Synthetic text data | Private | <https://doi.org/10.3390/ai7020069> |
| CARE-AD: a multi-agent large language model framework for Alzheimer’s disease prediction using longitudinal clinical notes | [20] | Observational study | LLaMA 3.1 | VHA longitudinal EHR | Electronic health record text | Restricted | <https://doi.org/10.1038/s41746-025-01940-4> |
| Predicting dementia from spontaneous speech using large language models | [21] | Observational study | GPT-3 | ADReSSo public speech transcripts | Speech and text | Public | <https://doi.org/10.1371/journal.pdig.0000168> |
| HoloDx: knowledge- and data-driven multimodal diagnosis of Alzheimer’s disease | [22] | Observational study | HuatuoGPT, GPT-4o | ADNI and four other datasets plus local cohorts | Medical imaging, clinical data, and multimodal information | Public and Restricted | <https://doi.org/10.1109/TMI.2025.3594364> |
| A large language model-based self-learning and critical agent framework for multimodal Alzheimer’s disease diagnosis | [23] | Observational study | GPT-4 | ADNI database | Imaging, clinical data, and biomarkers | Public | <https://doi.org/10.1037/neu0001079> |
| AI approaches for phenotyping Alzheimer’s disease and related dementias using electronic health records | [24] | Observational study | Me-LLaMA, Mistral | De-identified EHR notes from the MUSC research repository | Electronic health record text | Restricted | <https://doi.org/10.1002/trc2.70089> |
| Human and large language model judgments of cognitive impairment from language: an explainable artificial intelligence approach | [25] | Observational study | GPT-4o, LLaMA 3 | Pitt corpus | Speech and text | Public | <https://doi.org/10.1002/dad2.70248> |
| A GPT-4o-Powered Framework for Identifying Cognitive Impairment Stages in Electronic Health Records | [26] | Observational study | GPT-4o | MGB system EHR and memory clinic notes | Electronic health record text | Restricted | <https://doi.org/10.1038/s41746-025-01834-5> |
| Integrating fine-tuned LLM with acoustic features for enhanced detection of Alzheimer’s disease | [27] | Observational study | Llama2, Mistral-7B | ADReSSo 2021 | Speech and acoustic features | Public | <https://doi.org/10.1109/JBHI.2025.3566615> |
| Multimodal LLM for enhanced Alzheimer’s disease diagnosis: interpretable feature extraction from MMSE data | [28] | Observational study | Qwen-VL-Max；Qwen-Audio-Turbo | Multimodal MMSE data from the First Affiliated Hospital of Chongqing Medical University | Text, speech, and cognitive scores | Restricted | <https://doi.org/10.1016/j.exger.2025.112812> |
| CLONE: synthetic guideline-based clinical reasoning with large language models for early diagnosis of mild cognitive impairment | [29] | Observational study | LLaMA 3.3, Phi-4 | Hanyang University Hospital cohort | Clinical text and diagnostic information | Restricted | <https://doi.org/10.1145/3706599.3720111> |
| Data-driven analysis of Alzheimer’s disease classification using LLaVA-Med: a large language model approach | [30] | Observational study | LLaVA-Med | ADNI, OASIS, and AB-MRI datasets | MRI medical imaging | Public | <https://doi.org/10.1109/AICCSA63423.2024.10912607> |
| Enabling few-shot Alzheimer’s disease diagnosis on biomarker data with tabular LLMs | [31] | Observational study | Qwen2.5, GPT-4.1-mini | ADNI QT-PAD biomarker dataset | Structured biomarker data | Public | <https://doi.org/10.1145/3765612.3767229> |
| Alzheimer’s disease recognition from spontaneous speech using large language models | [32] | Observational study | GPT-4, GPT-3.5 | 237 recorded speech samples from ADReSSo | Speech and text | Public | <https://doi.org/10.4218/etrij.2023-0356> |

Note: Public indicates that the dataset is openly accessible; Restricted indicates that access to the dataset requires application, authorization, or institutional approval; Private indicates that the dataset is unavailable to external researchers; Public and Restricted indicates that the study simultaneously used publicly accessible datasets and restricted-access clinical datasets. AD: Alzheimer’s disease; LLMs: large language models; MCI: mild cognitive impairment; EHR: electronic health record; MMSE: Mini-Mental State Examination; EEG: electroencephalography; MRI: magnetic resonance imaging; ADReSS: Alzheimer’s Dementia Recognition through Spontaneous Speech; ADReSSo: Alzheimer’s Dementia Recognition through Spontaneous Speech Challenge; ADNI: Alzheimer’s Disease Neuroimaging Initiative; OASIS: Open Access Series of Imaging Studies; DementiaBank: DementiaBank language research database; PROCESS: database for speech and language assessment of cognitive impairment; Pitt Corpus: Pittsburgh University dementia speech corpus; VHA: Veterans Health Administration; MGB: Mass General Brigham healthcare system; MUSC: Medical University of South Carolina; AUC: area under the curve; ROC-AUC: area under the receiver operating characteristic curve; PR-AUC: area under the precision–recall curve; RMSE: root mean square error; MSE: mean squared error; LoRA: Low-Rank Adaptation; DOI: digital object identifier.
